# Glucose Dysregulation as a Driver of Autoimmune Mimicry, Inflammation, and Psychiatric Symptoms: A Narrative Review

**DOI:** 10.3390/biology15131094

**Published:** 2026-07-07

**Authors:** Jacob Warner-Palacio, Hannah Hunsaker, Amanda McKenna, Brigita Budginas, Levi Fridriksson, Alexander Tam, Christina Nelson, David Sant, Kyle B. Bills

**Affiliations:** Department of Research, Noorda College of Osteopathic Medicine, 2162 S 180 E, Provo, UT 84606, USA; do27.jswarner@noordacom.org (J.W.-P.); do27.hkhunsaker@noordacom.org (H.H.); do27.agmckenna@noordacom.org (A.M.); do27.brbudginas@noordacom.org (B.B.); do27.lgfridriksson@noordacom.org (L.F.); alexanderxtam@gmail.com (A.T.); christina.nelson@noorda.edu (C.N.); david.sant@noorda.edu (D.S.)

**Keywords:** glucose dysregulation, anxiety, depression, autoimmune, very low-carbohydrate diet, ketogenic diet, metabolism

## Abstract

Background: Glycemic control influences a wide range of psychiatric and physiological symptoms. Many psychiatric conditions are linked to poor glucose regulation. Anti-nuclear antibodies (ANAs), widely used to screen for autoimmune disease, may sometimes reflect transient metabolic immune activation rather than chronic pathology. Evidence suggests that glucose dysregulation, including glycemic variability, reactive hypoglycemia, and postprandial spikes, can trigger systemic inflammation, neuropsychiatric symptoms, and temporary autoantibody production. This review explores how these metabolic phenomena complicate diagnosis and may contribute to misclassification or overtreatment. Methods: We conducted a narrative literature review prioritizing studies on immune activation, psychiatric outcomes, metabolic measures (including continuous glucose monitoring), and therapeutic interventions. Articles from the last ten years were emphasized, with older foundational studies included when relevant. Results: Metabolic instability drives immune dysregulation through macrophage polarization, mitochondrial dysfunction, oxidative stress, and impaired clearance of apoptotic debris. Glycemic variability correlates with increased inflammatory cytokines, autonomic dysfunction, and psychiatric symptoms such as anxiety, depression, and cognitive impairment. Emerging evidence from small studies suggests that correcting glucose abnormalities may reduce metabolic dysfunction and improve neuropsychiatric symptoms. Conclusions: Recognizing glucose dysregulation as a contributor to autoimmune-like and psychiatric symptoms may refine diagnostic and therapeutic approaches. Incorporating a dynamic holistic approach, which in practice includes metabolic assessments, dietary interventions, use of continuous glucose monitoring, and history of blood sugar instability, into rheumatologic and psychiatric evaluations could improve diagnostic precision, reduce unnecessary immunosuppression, and enhance patient outcomes.

## Simple Summary

Some of the most difficult cases to diagnose involve patients presenting with generic symptoms like fatigue, anxiety, depression, pain, or brain fog, in part because they have so many different causes. Doctors commonly test for antibodies that can indicate autoimmune diseases, but these antibodies can also appear in normal populations, or even when the body is under stress from unstable blood sugar. New research shows that swings in blood sugar, even those in people without diabetes, can cause inflammation, stress on the brain, and temporary immune reactions that look like autoimmune problems. This indicates that some patients with blood sugar instability may be misdiagnosed with autoimmune disease. In this review, we examined scientific studies on how blood sugar fluctuations affect the immune system and the brain, and how this can produce symptoms that mimic autoimmune or psychiatric conditions. We also looked at approaches such as dietary changes and continuous blood sugar monitoring, which may help stabilize metabolism and improve symptoms. Understanding the link between blood sugar swings and immune or mental health symptoms may help doctors avoid unnecessary treatments and improve care. This information can empower patients and clinicians to consider metabolic health as an important part of diagnosing and managing complex symptoms.

## 1. Introduction

The interplay between metabolic health, immune function, and neurological regulation presents a compelling framework for understanding complex clinical presentations. Anti-nuclear antibodies (ANAs) are often regarded as biomarkers of systemic autoimmune disease, yet their presence can sometimes reflect transient immune activation rather than chronic pathology [1,2]. Several autoimmune diseases have ANA positivity as an associated biomarker with varying prevalence, including Sjogren syndrome, inflammatory myopathies, autoimmune hepatitis, and autoimmune thyroiditis [3]. However, other autoimmune diseases have a much higher prevalence of ANA positivity, including Systemic Lupus Erythematosus (SLE) and systemic sclerosis, with a well-established clinical finding of an ANA prevalence of 95–99% and 90%, respectively [4,5].

In many cases, ANAs can represent a broad and non-specific laboratory finding, and even detailed nuclear pattern analysis may yield indeterminate or misleading results. ANA positivity occurs in approximately 20–30% of the general population, yet only a minority of these individuals are ultimately diagnosed with autoimmune disease [1,6]. Additionally, ANA positivity has increased over time in US populations [7].

The presence of ANAs has also been observed in 22–24% of patients with type 1 or type 2 diabetes, implicating metabolic dysfunction as a potential driver of autoantibody formation [8]. While these values appear comparable to ANA rates in the general population, the studies differ substantially in assay thresholds, age distribution, and exclusion criteria. Large population studies reporting rates of 20–30% ANA positivity include all titers ≥1:80 and also include older adults (who naturally exhibit higher ANA rates) [1], whereas diabetes cohorts often use stricter cutoffs and narrower demographics, often excluding those with autoimmune diseases, advanced age, or other confounding factors. When these variables are controlled, individuals with metabolic disease demonstrate a reliably higher prevalence of ANA positivity than matched controls [8]. These findings challenge the long-held and increasingly outdated assumption that ANA reactivity is synonymous with autoimmune pathology. Instead, transient ANA production may arise from metabolic or inflammatory mechanisms such as oxidative stress, impaired apoptotic clearance, and/or macrophage dysfunction [6,9,10]. This overlap complicates diagnosis and may lead to the overuse of immunosuppressive therapies in patients whose symptoms originate not from autoimmune dysfunction, but from reversible metabolic disturbances.

Glucose dysregulation (often manifesting as chronic hyperglycemia, hypoglycemia, or profound glycemic variability) exerts profound effects on nearly every physiological system [11]. Beyond its role in endocrine and cardiovascular pathology, glycemic instability contributes to immune activation and neuroinflammation, both of which are central to autoimmune and psychiatric disease mechanisms [12,13,14,15]. The hypothalamus serves as a critical integrator of these systems, regulating glucose metabolism, stress responses, circadian rhythms, and mood [16]. Disruption within the hypothalamus or hypothalamic–pituitary–adrenal axis (HPA-axis) functionality can precipitate widespread physiological changes that mimic autoimmune disease, including fatigue, pain, rashes, and mood instability [17,18]. Autoimmune diseases such as SLE and systemic sclerosis can also show metabolic dysregulation. Patients with SLE show higher fasting insulin and glucagon levels, whereas patients with systemic sclerosis show paradoxically increased whole-body insulin sensitivity [19,20].

This review synthesizes current literature on the mechanisms by which glucose dysregulation may contribute to the imitation of autoimmune symptomatology, including transient ANA positivity and psychiatric symptomatology. By examining the interplay among glycemic variability, immune clearance, and hypothalamic regulation, we aim to establish both a mechanistic and clinical framework for understanding metabolically induced autoimmune mimicry. Additionally, therapeutic implications, including the use of very low-carbohydrate diets (VLCD) and continuous glucose monitoring (CGM), may represent key aids in treating systemic changes resulting from glucose variability and ANA positivity.

## 2. Literature Search Strategy and Study Selection

Mechanistic, translational, and clinical evidence linking glucose dysregulation, immune activation (particularly ANA production), neuroendocrine dysfunction, and psychiatric outcomes was synthesized with the goal of integrating existing literature into a clinically useful model of metabolically induced autoimmune mimicry. For consistency, glucose dysregulation was defined to include chronic hyperglycemia, reactive or postprandial hypoglycemia, and glycemic variability (as measured by CGM metrics such as standard deviation [SD], coefficient of variation [CV], and time-in-range [TIR]). For the purposes of this review, autoimmune mimicry was defined as clinical or laboratory findings that resemble autoimmune disease but arise from metabolic or inflammatory mechanisms rather than primary autoimmunity. These include serologic mimicry (transient or low-titer ANA positivity, pattern changes, and elevated inflammatory and autoantibody markers including C-reactive protein [CRP] and erythrocyte sedimentation rate [ESR] in the absence of autoimmune disease), clinical mimicry (fatigue, arthralgia, rashes, or neurologic symptoms including migraine, cognitive dysfunction, and psychiatric symptoms), and mechanistic mimicry (oxidative stress, mitochondrial dysfunction, and macrophage polarization patterns resembling autoimmune activation, including neutrophil extracellular traps [NET] formation).

The primary database searched was PubMed/MEDLINE (via National Center for Biotechnology Information [NCBI]), with full-text access through PubMed Central [PMC] when available. Searches were conducted in June 2025, focusing on studies published 2015–2025, with older seminal mechanistic papers included when foundational to pathophysiology. Only English-language full texts were analyzed; translated versions of critical non-English abstracts were used when available.

Search terms combined controlled vocabulary and free text using Boolean logic. Core queries included:

- (“ANA” OR “Anti-Nuclear Antibodies”) AND (“diabetes” OR “type 1” OR “type 2”);
- (“glycemic variability” OR “glucose variability”) AND (“inflammation” OR “oxidative stress” OR “neuroinflammation”);
- (“reactive hypoglycemia” OR “postprandial hypoglycemia”) AND (“anxiety” OR “psychiatric”);
- (“continuous glucose monitoring” OR “CGM”) AND (“autoimmune” OR “ANA” OR “inflammation”);
- (“hyperglycemia” OR “high glucose”) AND (“macrophage dysfunction” OR “impaired clearance”);
- (“apoptotic clearance”) AND (“autoantibody” OR “autoimmunity”);
- (“ketogenic diet” OR “low carbohydrate diet” OR “fasting”) AND (“immune modulation” OR “neuroprotective” OR “psychiatric” or “mental health”);
- (“hypothalamus” OR “HPA axis”) AND (“glucose regulation” OR “mood disorder”);
- (“advanced glycation end-products” OR “AGEs”) AND (“immune activation” OR “inflammation”).

The inclusion criteria encompassed human studies (cohort, cross-sectional, case series, or trials) investigating ANA prevalence in metabolic disorders, glycemic variability and inflammatory or neurocognitive outcomes, CGM use in autoantibody-positive populations, and dietary or metabolic interventions (e.g., ketogenic/VLCD). While priority was given to human-subject trials, some mechanistic studies (in vitro or animal) were included when they provided insight into macrophage dysfunction, apoptotic clearance, or hypothalamic regulation. Excluded studies lacked relevant immune, metabolic, or psychiatric endpoints, were single-case reports (unless highly illustrative), or were non-English without translation.

An initial retrieval of ~1500 unique records yielded 95 full texts screened for eligibility, of which 69 met inclusion criteria, many of which were used to provide background or repetitive information on similar topics, animal studies, or which were cited the same information in a more recent publication. Extracted data included metrics of glycemic variability, immune endpoints (e.g., ANA, cytokines, macrophage assays), neuropsychiatric measures, and therapeutic outcomes. Findings were synthesized narratively to integrate mechanistic, clinical, and therapeutic perspectives.

## 3. Results

### 3.1. Glucose Dysregulation and Inflammatory/Neuroendocrine Consequences

Glucose dysregulation encompasses a spectrum of abnormalities in blood sugar levels, including hyperglycemia, hypoglycemia, and significant glycemic variability [21]. Glycemic variability differs from traditional measures of glycemic control in that it captures rapid fluctuations in glucose levels over time, rather than averaged values or proxy measurements of end products, as measured with hemoglobin A1c (HbA1c). CGM enables high-resolution assessment of peripheral glucose dynamics on the scale of minutes, providing insight into metabolic instability that is not detectable through conventional measures, including HbA1c, which reflects the mean glycemia over weeks to months [12]. As such, CGM-derived variability metrics offer a more physiologically relevant window into metabolic stress, particularly in individuals without overt diabetes.

Additionally, glucose dysregulation represents a central mechanism linking metabolic instability to systemic inflammation, immune activation, and neuropsychiatric dysfunction [12,16,18,22,23,24,25]. Beyond the classic definitions of diabetes and insulin resistance, emerging evidence highlights that fluctuations in blood glucose, even fluctuations within non-diabetic ranges, can induce oxidative stress, impair mitochondrial function, and dysregulate immune signaling [12,21]. These effects occur independently of sustained hyperglycemia and highlight glycemic variability as a distinct pathophysiologic stressor. Elucidating the cellular and systemic pathways through which glycemic variability promotes inflammation provides critical insight into how metabolic disturbances may mimic, precipitate, or exacerbate autoimmune and psychiatric conditions.

#### 3.1.1. Clinical Manifestations of Chronic Glucose Dysregulation

Poor glycemic control has long been linked to oxidative stress, vascular dysfunction, and cognitive decline [12]. However, emerging evidence indicates that glycemic variability itself, not just sustained hyperglycemia, induces endothelial inflammation and oxidative injury [13,14,15,26]. Endothelial cells express a receptor for advanced glycation end products (RAGE), and recurrent hyperglycemic spikes increase oxidative signaling that promotes microvascular damage and blood–brain barrier permeability, potentially implicating metabolic stress in neurologic symptomatology [10]. Experimental models comparing stable versus oscillating glucose levels in both diabetic and non-diabetic participants found that repeated glucose fluctuations led to higher oxidative stress and greater endothelial dysfunction than single, acute hyperglycemic episodes [12]. These findings suggest that variability, rather than absolute glucose elevation alone, may be a primary driver of vascular and neurologic injury. Further, glycemic variability and the resultant oxidative and neuroinflammatory stress are increasingly implicated in migraines [27,28], mood instability, and psychiatric disorders [13,14,15,26,28]. The connection between metabolic and neuroimmune pathways suggests that chronic glucose dysregulation may contribute to psychiatric symptomatology by promoting neuroinflammation, disrupting neurotransmitter synthesis, and altering hypothalamic regulation of stress and energy homeostasis.

#### 3.1.2. Metabolic and Clinical Consequences of Acute and Chronic Hypoglycemia

Hypoglycemia can elicit oxidative and inflammatory stress through impaired mitochondrial bioenergetics [12,13,17]. Experimental models demonstrate that repetitive, mild hypoglycemia increases protein carbonylation, lipid peroxidation, and the expression of redox-related genes [17], while human studies show elevated inflammatory markers such as C-reactive protein and urinary free 8-isoprostoglandin F2α (8-iso PGF2α) [12].

Beyond classical metabolic effects, hypoglycemia is increasingly recognized as a potential trigger for systemic oxidative and inflammatory stress [29]. Experimental induction of hypoglycemia in humans has been shown to elevate stress proteins and markers including urinary isoprostanes and high-sensitivity C-reactive protein (hs-CRP) up to 24 h after the event [30]. This may indicate that metabolic stress from hypoglycemia can lead to oxidative injury and vascular inflammation, even if only transiently, following episodes of hypoglycemia [30]. Additionally, a more recent study from Verhulst et al., 2022 demonstrated that hypoglycemia increases circulating lymphocytes and monocytes, as well as enhances monocyte pro-inflammatory cytokine release and raises multiple inflammatory proteins over a week [31]. This indicates sustained hypoglycemia may produce prolonged immune activation, not only an acute response [31,32].

These episodes of hypoglycemia, whether induced from post-prandial reactive hypoglycemia, iatrogenic causes, or systemic disorders, may produce lasting inflammatory effects that can affect many immune and oxidative markers [32]. Hypoglycemia triggers counter-regulatory stress responses including catecholamine release, which increases inflammatory cytokines and endothelial dysfunction [12,22,33]. Chronic or repeated hypoglycemia may contribute to neuroendocrine stress signaling and sympathetic activation [32].

These stress states are recognized drivers of neuronal injury, with oxidative stress, neuroinflammation, and reactive oxygen species (ROS) production linked to mitochondrial dysfunction and cellular damage [12]. Additionally, this oxidative stress has been identified as a unifying mechanism across many migraine triggers, such as metabolic shifts including hypoglycemia [27,33]. Some studies indicate that oxidative stress may contribute to neurogenic inflammation, which may trigger migraine pathways including transient receptor potential ankyrin 1 (TRPA1) and calcitonin gene-related peptide (CGRP). Collectively, these findings indicate that hypoglycemia may serve as a biologically active stressor influencing immune, vascular, and neural function beyond simple glucose deprivation [33].

#### 3.1.3. Metabolic and Clinical Consequences of Acute and Chronic Glycemic Variability

Importantly, oscillating between hypo- and hyperglycemia amplifies reactive oxygen species (ROS) production beyond the effect of either condition alone, in part through recurrent activation of mitochondrial electron transport and nicotinamide adenine dinucleotide phosphate (NADPH) oxidase pathways [12]. Consequently, glycemic variability, typically marked by oscillations between hyper- and hypoglycemia, appears to induce greater oxidative and inflammatory stress than either condition alone [12,13,14,15].

Recurrent glycemic oscillations also influence innate immune activation. Although macrophage biology has historically been described using the binary M1/M2 framework, contemporary evidence demonstrates that macrophage activation exists along a metabolically driven spectrum [9,10,34,35]. In the setting of glycemic variability and excess free fatty acids (FFAs), macrophages adopt a metabolically activated phenotype characterized by enhanced glycolysis, impaired autophagy, defective efferocytosis, and sustained nuclear factor kappa-light-chain-enhancer of activated B cells (NF-κB) [6,10,25]. Unlike pathogen-driven macrophages, these metabolically activated macrophages show exaggerated responsiveness to RAGE signaling and accumulate uncleared apoptotic debris, fostering chronic inflammation and contributing to autoantibody formation [6,10]. This spectrum-based model more accurately reflects the macrophage phenotypes observed in diabetes, obesity, and glycemic variability, and provides a mechanistic bridge between metabolic dysfunction and autoimmune-like clinical presentations [6,10,25].

#### 3.1.4. Metabolic and Clinical Consequences of Acute and Chronic Hyperglycemia

Prolonged hyperglycemia, even in the absence of marked glycemic variability, promotes the formation of advanced glycation end products (AGEs), which covalently modify proteins and nucleic acids. These altered molecules are poorly recognized by macrophage clearance receptors, leading to impaired efferocytosis and accumulation of cellular debris. Concurrently, hyperglycemia directly impairs macrophage phagocytic capacity, further delaying debris clearance and prolonging antigen exposure [10,25].

Persistence of modified nuclear antigens in the extracellular milieu facilitates immune recognition and autoantibody production, contributing to autoimmune-like responses. In parallel, AGEs engage RAGE, activating NF-κB signaling, increasing ROS production, and sustaining cytokine release, even after normalization of glucose levels, and thereby perpetuating chronic inflammation [10].

While diabetes mellitus remains a common cause of sustained hyperglycemia, other factors including hormonal dysregulation (elevated cortisol or catecholamines), acute injury, surgery, oxidative stress, medication, and psychological stress can induce prolonged hyperglycemia independent of pre-existing intrinsic insulin dysfunction [24,28]. Sustained hyperglycemia can sensitize macrophages toward the M1 phenotype (using the historic classification), heightening ROS production and cytokine release. When this sensitization is compounded by glycemic oscillations, the AGE-RAGE axis remains chronically engaged, maintaining NF-κB activation and impairing autophagy, which reduces phagocytic capacity and disrupts the balance between M1 and M2 (or rather, pro-inflammatory vs. anti-inflammatory) activity, ultimately perpetuating oxidative stress, chronic inflammation, and tissue injury [25].

#### 3.1.5. Pathophysiologic Effects of Glucose Dysregulation on Neuroimmune and Endocrine Networks

Chronic glucose dysregulation also perturbs neuroimmune and endocrine signaling, particularly through alterations in HPA axis function and cytokine-mediated communication between the nervous and immune systems [17]. Glycemic variability may act as a physiological stressor, repeatedly activating hypothalamic glucose-sensing neurons and promoting dysregulated corticotropin-releasing hormone (CRH) signaling [11,22,24]. This results in exaggerated or prolonged cortisol release, impaired diurnal rhythmicity, and reduced feedback inhibition at the level of the hippocampus and hypothalamus, thereby sensitizing the stress response system to subsequent metabolic or psychological challenges [11,12,36].

In parallel, fluctuating glucose levels exert direct effects on central nervous system immune cells. Recurrent hyperglycemic and hypoglycemic excursions promote microglial priming, characterized by heightened responsiveness to inflammatory stimuli, increased production of proinflammatory cytokines, and amplified reactive oxygen species (ROS) generation [10,12,25]. This primed microglial state disrupts synaptic plasticity, impairs neurogenesis, and alters glutamatergic and monoaminergic neurotransmission, linking metabolic instability to cognitive dysfunction, mood dysregulation, and heightened stress sensitivity [12,21,36,37,38].

These neuroimmune alterations further interact with peripheral inflammatory signaling. Circulating cytokines and AGE-modified molecules cross or signal through a compromised blood–brain barrier, reinforcing central inflammation and perpetuating hypothalamic–pituitary–adrenal (HPA) axis activation [12,16,18,21,22,36,38]. The resulting feed-forward loop between metabolic stress, neuroinflammation, and endocrine dysregulation contributes to emotional lability, fatigue, sleep disturbance, and anxiety-related symptomatology frequently observed in chronic metabolic disorders [12,13,14,15,18,26,39]. Collectively, ROS accumulation, AGE-RAGE signaling, impaired autophagy, and immune activation integrate metabolic instability with neuroendocrine dysfunction, bridging mechanistic pathways traditionally treated as separate domains.

### 3.2. Autoimmune Activation in Metabolic Dysfunction: ANA Formation and Autoimmune Mimicry

Anti-nuclear antibodies and autoimmune mimicry represent two interrelated immunologic phenomena that complicate the differential diagnosis of systemic and metabolic disorders. ANAs are a hallmark of autoimmune diseases such as systemic lupus erythematosus (SLE) and mixed connective tissue disease (MCTD), yet they can also appear transiently in metabolic disturbances or even healthy individuals [40]. Similarly, autoimmune mimicry describes conditions that imitate autoimmune disease both clinically and serologically, often arising from non-autoimmune mechanisms such as infection or metabolic stress [41]. For example, clinical mimicry may present as “autoimmune-like” symptoms due to metabolic causes, whereas serological mimicry may present as ANA positivity on serology in the absence of disease [7]. Emerging mechanistic evidence suggests that oxidative stress, impaired apoptotic clearance, and formation of modified autoantigens during hyperglycemia can transiently activate adaptive immunity, providing a critical framework for distinguishing true autoimmune pathology from reversible metabolic conditions.

#### 3.2.1. Metabolic and Immunologic Mechanisms Driving Anti-Nuclear Antibody Formation

ANAs represent a broad spectrum of autoantibodies directed against nuclear antigens including DNA, histones, and RNA-binding proteins. Their presence is strongly associated with autoimmune disease, yet studies show that ANA positivity can occur in healthy individuals or in transient inflammatory states [40]. Under physiological conditions, cellular debris is efficiently cleared by “natural autoantibodies” and macrophages through non–antigen-presenting pathways. When clearance is impaired, apoptotic material is instead phagocytosed by antigen-presenting cells, triggering an adaptive immune response and subsequent autoantibody production. Murine models demonstrate that ANAs may emerge following inoculation alongside protective forms of “natural” autoimmunity, suggesting a context-dependent role [40]. Regulation of these antibodies appears to depend on circulating autoantigen concentrations and varies substantially between individuals, making immunologic screening complex and often nonspecific.

Metabolic disturbances, particularly recurrent hyperglycemia and glycemic variability, increase oxidative modification of nuclear proteins, creating neo-epitopes that are more likely to be recognized as foreign [25]. Metabolic stress also disrupts macrophage homeostasis by altering inflammatory signaling, impairing autophagy, and reducing phagocytic clearance capacity. Accelerated macrophage apoptosis increases antigenic burden and has been shown to exacerbate autoantibody formation and end-organ damage such as nephritis [35]. Additionally, hyperglycemia drives the formation of AGEs, which bind to the RAGE receptor on immune cells and amplify NF-κB–mediated inflammatory signaling, further augmenting nuclear antigen presentation.

Delayed clearance of apoptotic debris, whether from phagocytic dysfunction or excessive cellular turnover, prolongs exposure to nuclear antigens and drives chronic immune activation [42]. Similarly, persistent NETs can potentiate autoantibody formation by increasing circulating nuclear material. Together, these mechanisms illustrate how metabolic stress may transiently elevate ANA production without a primary autoimmune etiology.

Epidemiological data supports this association. Litwińczuk-Hajduk et al. [8] reported ANA positivity in 24% of individuals with type 1 diabetes and 22% with type 2 diabetes, compared to none in healthy controls. In a large cohort study, Ge et al. [2] identified ANA positivity in 7.09% of 14,186 healthy participants, with positive individuals more likely to exhibit metabolic abnormalities. While ANA positivity is not uncommon in the general population, its prevalence is notably higher among those with glucose dysregulation, supporting the hypothesis that oxidative inflammation, AGE-mediated signaling, and impaired antigen clearance may transiently induce ANA formation [2,8].

#### 3.2.2. Metabolic-Induced Autoimmune Mimicry and Serologic Cross-Reactivity

Autoimmune mimicry refers to immune responses against foreign or altered self-antigens that generate cross-reactivity with host tissues. Microbial antigens, chemically modified proteins, or metabolic byproducts can all provoke such cross-reactive responses, producing both humoral and cellular findings consistent with autoimmune disease. In the context of glucose dysregulation, post-translational modifications of self-proteins, oxidative damage to nuclear structures, and persistent NETosis increase the likelihood that immune receptors mistake transient metabolic debris for autoimmune targets [25]. In these cases, both serological mimicry and clinical mimicry may arise. In laboratory findings, serological mimicry may show ANA positivity in the absence of disease, indicating that many healthy adults may show transient increases in serum ANA levels and other autoimmune markers despite the absence of clinical symptoms [2,40]. However, some populations may demonstrate clinical mimicry, or autoimmune-like symptoms that may be tied to metabolic causes [8,43]. Consequently, serological mimicry may occur, leading to laboratory findings that can resemble metabolic autoimmune pathology or a pseudo-inflammatory state despite the absence of a primary autoimmune mechanism [40].

This diagnostic overlap presents a significant challenge. Previously cited studies indicated ANA positivity in 22–24% of patients with diabetes mellitus and up to 25% of healthy individuals, emphasizing that ANA detection alone is insufficient to diagnose autoimmune disease [41]. Notably, these findings differ from the study by Litwińczuk-Hajduk et al. [8], which identified no ANA positivity among healthy controls. Similar variability in ANA prevalence has been previously described by Grygiel-Górniak et al., who noted that highly sensitive indirect immunofluorescence assays may detect ANA positivity in up to 25–40% of otherwise healthy individuals, further emphasizing the limited specificity of ANA testing in isolation [44]. Grygiel-Górniak et al. further concluded that ANA positivity alone is insufficient for diagnosing autoimmune disease given the substantial prevalence of low-titer positivity reported in healthy populations [44]. Clinical context must therefore be integrated with metabolic history, exposure to glycemic variability, and improvement following metabolic stabilization to avoid misinterpretation. Recognizing how metabolic dysfunction can mimic autoimmune pathology, both clinically and serologically, is essential to prevent misdiagnosis and inappropriate immunosuppressive therapy. Future research is needed to determine whether metabolic stabilization reverses autoantibody titers, mitigates NET persistence, and normalizes macrophage phenotype, and to develop diagnostic criteria capable of distinguishing metabolic-induced immune activation from true autoimmune conditions.

### 3.3. Hypothalamic and Psychiatric Consequences of Glucose Dysregulation

**Figure 1:**
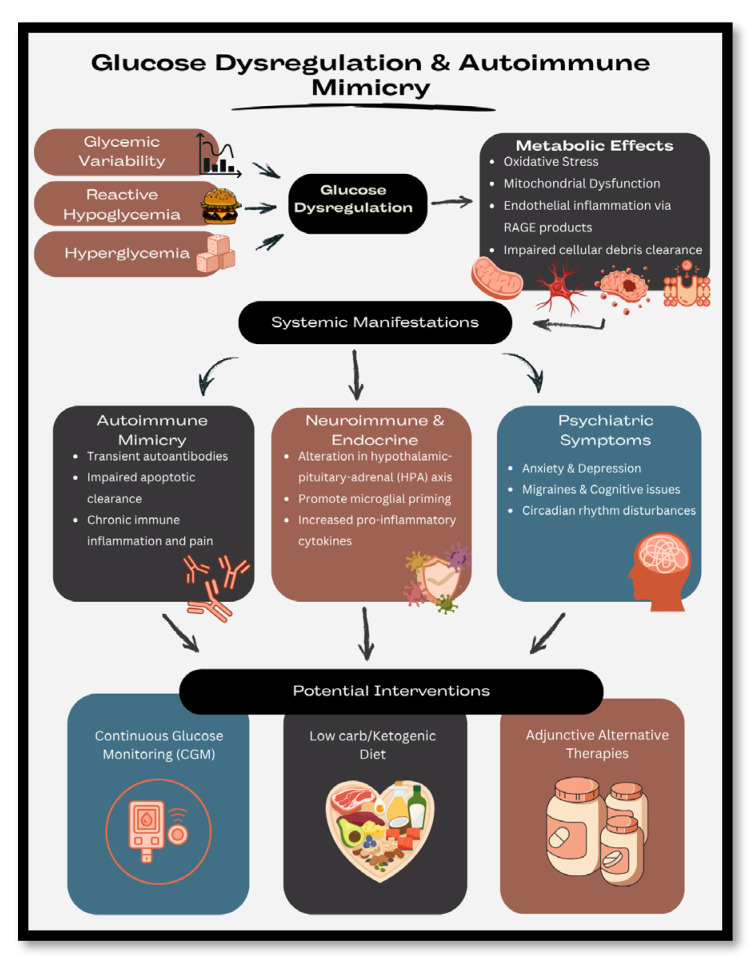
Overview of types of glucose dysregulation, effects on metabolism, and the systemic responses following metabolic alterations from glucose dysregulation.

Glucose dysregulation has systemic consequences that are not limited to metabolic and autoimmune pathology [12]. The brain plays a central regulatory role in glucose regulation (primarily through hypothalamic control), and fluctuations affect both psychiatric and neurologic processes [36,45]. However, both peripheral and central dysfunction can disrupt euglycemia and identifying root causes can pose diagnostic challenges [13]. Despite these challenges and considering the potential systemic and varied impact of dysregulation, it is imperative to consider the possibility of glucose dysregulation in the case of atypically presenting or treatment-resistant neurologic or psychiatric diagnoses [12,26,28]. Figure 1 illustrates the proposed interplay of glucose dysregulation, systemic effects, and proposed treatments.

#### 3.3.1. Hypothalamic Dysregulation and Psychiatric Manifestations

The hypothalamus plays a central role in coordinating glucose metabolism, immune regulation, and stress responses. Hypothalamic neurocircuits interact with the mesolimbic reward system where changes in glucose availability alter dopaminergic activity in the ventral tegmental area [36]. The hypothalamus also regulates immune responses via glucocorticoid signaling, crucial for balancing anti-inflammatory and immunosuppressive responses [45]. When dysregulated, whether due to trauma, hormonal imbalances, or metabolic stress, it can trigger systemic effects that influence endocrine function, metabolism, appetite, autonomic regulation, and mental health [16].

Glucose dysregulation may also influence Agouti-related peptide (AgRP) and Pro-Opiomelanocortin (POMC) neurons in the hypothalamic arcuate nucleus (ARC) [46]. Many peripheral signals, particularly leptin, insulin, and glucose, converge on POMC and AgRP or Neuropeptide-Y (NPY) neurons in the arcuate nucleus to control energy homeostasis [46]. This interaction can impair the ability of the melanocortin system to maintain appetite and energy homeostasis. POMC anorexigenic signaling predominates in the fed state while AgRP orexigenic signaling predominates in the fasting state [47]. AgRP dysfunction may specifically contribute to glucose dysregulation, as acute activation of AgRP neurons can induce systemic insulin resistance through reprogramming of gene expression in brown adipose tissue [48]. This suggests that AgRP neuron dysfunction in the context of metabolic disease may simultaneously drive hyperphagia and impair peripheral glucose handling [48].

In rats, chronic hyperglycemia induces glucose insensitivity in POMC neurons leading to impaired calcium responses to high blood glucose [49]. Specifically, disrupting glucose sensing in POMC neurons impairs whole-body glucose tolerance, and POMC neurons become defective in obese mice on a high-fat diet [49]. POMC mRNA expression is also reduced, as well as glucokinase expression, in the ARC, collectively resulting in hyperphagia. Additionally, correcting hyperglycemia with the anti-diabetic agent miglitol restored POMC expression and ameliorated hyperphagia, suggesting a causal link between glucose dysregulation and POMC neuron dysfunction [50]. These findings suggest a significant role of glucose dysregulation in behavioral changes.

The connection between glucose fluctuations and mental health is well-documented, with research linking glycemic instability to mood disturbances, anxiety, and depression [13,14,15]. Chronic hyperglycemia and hypoglycemia are associated with alterations in neurotransmitter balance, increased oxidative stress, and activation of inflammatory pathways, suggesting their importance in psychiatric conditions [12]. This bidirectional influence between metabolic regulation and mental health underscores the necessity of considering glucose homeostasis when addressing psychiatric conditions, particularly in patients with atypical or treatment-resistant symptoms. 

Psychiatric medications are a mainstay of modern treatment and can affect glucose homeostasis. Antipsychotics and antidepressants have a well-known association with weight gain and the associated cardiometabolic consequences; more recent evidence suggests that treatment with antipsychotics is associated with glucose dysregulation, regardless of weight [51]. The role of antidepressants in glucose homeostasis is more complex [52]. The physiological effects differ vastly based on the class of antidepressants [52]. The Serotonin-Norepinephrine Reuptake Inhibitor (SNRI) duloxetine has been shown to increase total cholesterol and glucose concentration; no other antidepressant has been definitively implicated in glucose regulation [52]. The concomitant use of multiple classes of psychiatric medication has yielded mixed results and warrants further investigation [51]. The role of psychiatric medication in glucose homeostasis and hypothalamic dysregulation should be considered in the development of a treatment plan.

Beyond its metabolic effects, hypothalamic dysfunction can have widespread consequences for homeostasis and can contribute to endocrine disorders, insulin resistance, circadian rhythm disturbances, autonomic nervous system dysfunction, and heightened stress responses via the HPA axis [16,36]. Additionally, evidence suggests a link between childhood trauma and autoimmune-like presentations, with trauma exposure increasing the risk of systemic inflammation and conditions such as systemic lupus erythematosus (SLE) [53]. Trauma-mediated changes to HPA axis tone may also sensitize hypothalamic networks, compounding both metabolic and psychiatric vulnerability [45].

#### 3.3.2. Neuropsychiatric Effects of Glucose Dysregulation

Metabolic dysfunction has been related to both systemic and neuroinflammation through multiple signaling pathways. A notable proportion of patients diagnosed with metabolic dysfunction present with comorbid psychiatric illness [26]. Specifically, type 2 diabetes mellitus (T2DM) is significantly associated with comorbid depressive disorder [54]. One analysis demonstrated that 19% of study subjects with T2DM had also been diagnosed with depression [54]. Recent studies suggest that individuals with metabolic disorders and inflammatory markers often suffer from atypical depression symptoms such as hyperphagia, weight gain, and leaden paralysis [26]. Atypical depression is often more difficult to treat; this emerging evidence suggests that testing for and treating metabolic symptoms to mitigate neurogenic inflammation in addition to treating psychiatric symptoms may allow for a better overall treatment response [12,13,14,15,26].

Lactate, produced during anaerobic metabolism, is used in the brain during times of glucose depletion [38]. Lactate infusion has been associated with the onset of panic attack-like symptoms such as fear of dying, sweating, shaking, and nausea [38]. Research has demonstrated that individuals suffering from panic disorder tend to accumulate higher levels of lactate in specific regions of the brain that are sensitive to sensory stimulation, though these mechanisms are poorly understood [38]. Individuals with glucose dysregulation may unexpectedly enter states of hypoglycemia and are thus forced to shift to anaerobic metabolism more frequently [13,14,15,53]. The increased frequency of anaerobic metabolism could explain the higher incidence of anxiety and panic-like symptoms in individuals with metabolic dysfunction and glucose dysregulation.

The brain is considered an insulin-sensitive organ and, as discussed previously, is highly sensitive to metabolic disturbance and fluctuations in glucose [28]. Although the mechanisms of migraine are not fully understood, research has demonstrated a link between altered insulin sensitivity, metabolic dysfunction, and migraine pathophysiology [28]. The connection between metabolic dysfunction and migraine implicates interconnected pathways like transient receptor potential vanilloid 1 (TRPV1), calcitonin gene-related peptide [CGRP], and neurogenic inflammation. Incidentally, these pathways are pharmacologically targeted in modern migraine treatments. In glucose dysregulation, neurogenic inflammation due to episodes of hyper- and hypoglycemia could lead to the onset of migraine via a similar mechanism [27]. Consequently, stabilizing glucose levels could potentially offer a viable treatment alternative to some of these neurologic and psychiatric presentations.

Additionally, astrocytes, which serve as primary glucose sensors in the brain, function as intermediaries between the peripheral metabolic environment and neuronal function. GLUT-1 transporters allow astrocytes to actively detect and respond to fluctuations in brain parenchymal glucose levels, and circulating hormones including leptin and glucagon-like peptide-1 (GLP-1) aid in regulating energy balance [55]. Hyperglycemia can also affect astrocytes and microglia. Astrocytes have been implicated in the neuroinflammatory response via the production of proinflammatory cytokines [56]. A key downstream mechanism, the astrocyte-neuron lactate shuttle (ANLS), allows astrocytes to take up circulating glucose, metabolize it to lactate, and transfer lactate to neurons for subsequent use as substrate in synaptic activity [57]. Disruption of this shuttle through glucose deprivation, hyperglycemia, or other forms of parenchymal stress can depress hippocampal synaptic transmission through calcium elevation and inhibition of excitatory neurotransmission [58].

Hyperglycemia is common following traumatic brain injury or ischemic stroke; astrocytes have been shown to differentiate into proliferative astrocytes in response to hyperglycemia [56]. Hyperglycemia due to metabolic dysfunction has been shown to exhibit similar proinflammatory properties, and this could contribute to neurologic and psychiatric presentations related to metabolic dysfunction and glucose dysregulation [55,58].

### 3.4. Therapeutic Targets and Considerations

Therapeutic approaches aimed at stabilizing glucose metabolism may mitigate the broad systemic consequences of dysregulation, including neuroinflammation, psychiatric symptomatology, and immune activation. Emerging evidence suggests that dietary modification (including a ketogenic diet, Mediterranean diets, and intermittent fasting), CGM, and adjunctive alternative treatments can support metabolic homeostasis, improve cellular energy efficiency, and reduce inflammatory burden [38,59,60]. By identifying glucose variability early, guiding individualized nutritional strategies, and addressing autonomic and structural contributors, clinicians can intervene before metabolic disturbances are misinterpreted as primary autoimmune disease. Taken together, these modalities emphasize a patient-centered, holistic framework that targets underlying physiologic imbalance rather than isolated symptoms.

#### 3.4.1. Diagnostics, Monitoring, and Therapeutic Strategies

Continuous glucose monitoring has evolved from a tool primarily used in type 1 diabetes to a broader clinical and research instrument with potential diagnostic, preventive, behavioral, and metabolic applications. Continuous glucose monitoring systems measure interstitial glucose every 1–5 min, serving as a relatively accurate proxy for blood glucose. This provides comprehensive data on glucose excursions, variability, and time-in-range (TIR) that are not captured by sporadic fingerstick measurements or A1c alone. In addition to aiding in diagnostic expansion, CGM can have several downstream effects through behavioral changes following consistent monitoring that aid in glycemic management.

Systematic reviews and meta-analyses confirm that CGM use is associated with improved glycemic control, most notably reductions in HbA1c and increases in TIR [61]. In patients with type 2 diabetes, CGM intervention programs reduced HbA1c significantly (mean effect of roughly −0.37%) and were particularly effective in older adults and real-time CGM users [62]. Longitudinal observational data in type 1 diabetes also show significant reductions in A1c over one year of CGM use, with corresponding increases in TIR [63].

Beyond traditional diabetes management, CGM metrics such as glycemic variability, TIR, and time above range are emerging as predictive markers for disease progression and complications. Variability and low TIR have been linked to microvascular and macrovascular complications, including retinopathy, albuminuria, and cardiovascular disease risk [64]. In nondiabetic cohorts, CGM-derived metrics correlate with insulin resistance and cardiometabolic profiles and may reveal patterns that traditional fasting glucose measures miss [23]. Additionally, CGM-based feedback alone can improve glycemic outcomes and support behavior change in both diabetic and non-diabetic populations [61]. Individuals receiving CGM feedback showed modest reductions in HbA1c and increased TIR compared with controls [65]. Further, CGM interventions often improve dietary and physical activity behaviors, suggesting CGM’s utility as a biofeedback tool that can engage users in self-management based on real-time data [66].

Continuous glucose monitoring data also interacts with psychological and self-management factors. Trials integrating CGM into structured self-care education demonstrate improvements in self-management adherence and reductions in anxiety and depression among adults with type 1 diabetes, suggesting psychosocial benefits in addition to metabolic improvements [62]. Continuous glucose monitoring represents a promising intervention that can provide both diagnostic and therapeutic benefits for patients managing multiple forms of glycemic variability, as well as potential psychological benefits whether from glycemic regulation, behavioral modification, or both.

#### 3.4.2. Dietary Interventions

##### Very Low-Carbohydrate Dietary Interventions

Very low-carbohydrate diets (VLCDs) are typically defined as diets that restrict carbohydrate intake to levels that are low enough to induce shifts in energy metabolism, often resulting in increased fat oxidation and, under sufficient restriction, ketosis [67,68]. Precise thresholds vary across studies, but most clinical research defines VLCDs as <50 g of carbohydrates per day or <10% of total daily energy from carbohydrates, often associated with physiological ketosis in many individuals [69]. For the treatment of epilepsy, according to consensus and clinical reviews, children are initially set at 10 g/day in classic ketogenic protocols, whereas adults and adolescents are set with a goal of 15–20 g/day, with allowance to increase to 25–30 g/day based on response and ketosis level [67,68].

Classic examples of VLCDs and related approaches include the classic ketogenic diet [67,68], modified Atkins diet [70,71,72], low glycemic index treatment [73], paleo [74,75,76], carnivore [77], and broadly, the very low-calorie ketogenic diet, among others. The ketogenic diet is a specific category of VLCD, and is characterized by a high-fat, moderate-protein, and low-carbohydrate approach (usually less than 50 g/day). The dietary prescription induces ketosis, a metabolic state in which fat serves as the primary energy source by mimicking metabolic starvation [78]. There are four types of ketogenic diets: standard, cyclical, targeted, and high protein, with the standard diet consisting of approximately 70% fat, 20% protein, and 10% carbohydrates being the most studied [79]. While other VLCDs may be employed for therapeutic management of some conditions, the ketogenic diet has been frequently utilized in conjunction with neurologic conditions, including treatment and management of seizure disorder [60]. Consequently, the ketogenic prescription method is often a clinical target when considering glucose dysregulation management.

This diet has been associated with stabilized blood glucose levels, reduced systemic inflammation, and enhanced mitochondrial function [39,60,80]. Specific ketone bodies, including β-hydroxybutyrate and acetoacetate, have protective properties such as mitochondrial efficiency improvement and oxidative stress reduction that minimize injury through mechanisms similar to calorie restriction [60]. Additionally, the permeability of the blood–brain barrier to ketones is increased through monocarboxylic acid transporters when the body is in a state of ketosis [60]. These neuroprotective properties are further supported by the promotion of glial formation in the hippocampus, which contributes to neuronal survival. In contrast to these documented outcomes, patient tolerability may be limited, with observational findings of hyperlipidemia, nephrolithiasis, and slowed growth [60].

The ketogenic diet has often been successfully used in conjunction with mental health disorders and suggests the potential benefits of glucose stabilization on psychiatric health [81]. Following a recent study, Brief Psychiatric Rating Scale scores have decreased by 32% in individuals with schizophrenia or bipolar disorder, and a recent clinical trial by Gao et al. showed a statistically significant reduction in PHQ-9 scores at 6 weeks for patients with treatment-resistant depression [82,83]. Because not all mental disorders can be directly linked to metabolic disruption, stratification of underlying cause may be a confounding factor limiting many studies investigating the ties between dietary intervention, metabolic disturbances, and mental health.

##### Mediterranean Dietary Interventions

While ketogenic interventions emphasize carbohydrate restriction and ketone production, the Mediterranean diet provides a more moderate, sustainable approach with substantial evidence for metabolic and cardiovascular health [59]. This dietary pattern prioritizes whole foods, particularly fruits, vegetables, legumes, whole grains, olive oil, fish, and nuts, while limiting red meat and processed foods. The high content of monounsaturated fats and polyphenols exerts potent anti-inflammatory and antioxidant effects, improving endothelial function and insulin sensitivity [59]. Studies link Mediterranean adherence to reduced risk of type 2 diabetes, cardiovascular disease, and cognitive decline, as well as improvements in depression and anxiety symptoms [59,84]. Unlike strict carbohydrate restriction, this diet allows greater flexibility, enhancing patient adherence and long-term metabolic balance. Incorporating elements of both Mediterranean and ketogenic strategies, such as low glycemic load, nutrient density, and healthy fats, may optimize individualized approaches to glucose stabilization and systemic inflammation reduction.

##### Fasting-Based Dietary Interventions

Fasting, a precursor to the ketogenic diet, was first documented in the early 20th century as a treatment for epilepsy [80]. In 1921, the ketogenic diet emerged as a more sustainable alternative, demonstrating similar efficacy in seizure management [80]. Both calorie restriction and ketosis result in decreased carbohydrate intake and increased ketone production. These combined effects have anticonvulsant and neuroprotective outcomes by enhancing mitochondrial stability and reducing oxidative damage [60]. The hypothalamus detects ketosis and increases HPA-axis tone, further contributing to metabolic and neurological stability [37].

##### Physiologic and Psychiatric Effects of Dietary Interventions

Dietary interventions that modify carbohydrate availability and metabolic fuel utilization exert diverse effects across neurologic, psychiatric, metabolic, and immune-mediated conditions. Among these, ketogenic and other very low-carbohydrate diets have received the greatest investigative attention, particularly in neurologic disease.

Ketogenic diets have been evaluated in a range of psychiatric conditions, including anxiety disorders, major depressive disorder, bipolar disorder, schizophrenia, autism spectrum disorder, and attention-deficit hyperactivity disorder [81]. Evidence from animal models, case series, and limited open-label human studies suggests potential benefits in anxiety and depressive symptoms [81]. A recent systematic review and meta-analysis of 50 studies showed that ketogenic diets have small-to-moderate improvements in depressive symptoms, with effects enhanced with ketone monitoring and in non-obese patients. Meanwhile, there is still a lack of evidence pertaining to anxiety, as more standardized protocols are needed [85]. A study from 2025 found that early onset type 2 diabetes was dramatically associated with increased risk of psychiatric illness development, including changes in Hazard Ratio (HR) of bipolar disorder (HR 4.17), depression (HR 3.97), anxiety (HR 3.76) and stress-related disorders (HR 3.35) [86]. Across psychiatric indications, interpretation is limited by small sample sizes, variable adherence, short intervention duration, and substantial methodological heterogeneity. At present, ketogenic interventions are not considered standard of care for psychiatric disorders, underscoring the need for adequately powered randomized controlled trials stratified by metabolic phenotype.

In contrast, the strongest clinical evidence for ketogenic dietary therapy remains in drug-resistant epilepsy [60,69,70]. Across pediatric and adult populations, approximately 50% of patients with refractory epilepsy achieve a ≥50% reduction in seizure frequency, with a subset experiencing near-complete seizure remission [69,80]. These improvements are frequently accompanied by gains in attention, alertness, and cognitive function, supporting a neuroprotective role beyond seizure suppression [60,80].The consistency of these findings has established ketogenic therapy as a recognized non-pharmacologic treatment option in refractory epilepsy.

Additionally, physiologic impacts on glucose levels represent a strong driving factor for prescriptive use. Among the listed interventions, the ketogenic diet has the most robust evidence for glycemic improvement. Meta-analyses of randomized controlled trials in overweight patients with Type 2 Diabetes Mellitus demonstrate significant reductions in HbA1c, fasting glucose, and insulin resistance, with some trials showing greater HbA1c reduction in ketogenic diet groups compared to standard diabetic diets [79]. A network meta-analysis comparing multiple dietary approaches found that ketogenic and low-carbohydrate diets produced significant reductions in HbA1c of approximately −0.73% and −0.69%, respectively, compared to control diets, with Mediterranean and low glycemic index diets demonstrating the strongest effects on fasting glucose reduction [87]. Fasting-based interventions, including intermittent fasting and time-restricted eating, similarly improve glycemic outcomes [88]. All four examined intermittent fasting regimens (2x weekly fasting, fasting-mimicking diet, time-restricted eating, and periodic fasting) outperformed conventional diet on blood glucose and insulin sensitivity [88].

Emerging evidence also supports the role of ketogenic and VLCD-based interventions in neurodegenerative disease. In Alzheimer’s and Parkinson’s disease, dietary ketosis has been associated with improved neuronal energy metabolism, enhanced mitochondrial function, and increased resistance to oxidative injury, contributing to functional resilience and slowed cognitive decline in some clinical and preclinical models [28,80,81,89]. Although long-term clinical outcome data remain limited, these findings suggest that impaired glucose utilization may represent a modifiable contributor to neurodegeneration.

Beyond neurologic disease, ketogenic and VLCD interventions have demonstrated therapeutic relevance in metabolic and immune-mediated conditions, including type 2 diabetes mellitus and autoimmune disorders [90]. Type 2 diabetes risk is increased with the co-diagnosis of 27 of 32 autoimmune diseases, and autoimmune disease risk is also elevated after type 2 diabetes diagnosis, which suggests a bidirectional relationship between inflammatory mechanisms rather than strictly lifestyle alone [91]. In multiple sclerosis models, ketogenic diets have been shown to reduce microglial and astrocytic activation while promoting oligodendrocyte maturation and myelin integrity, effects consistent with reduced neuroinflammation and improved cellular energetics [60,90]. Additional immune-modulatory effects have been observed in inflammatory conditions such as uveitis, where dietary ketosis has been associated with increased regulatory T-cell differentiation and attenuation of inflammatory T-cell responses [60,92].

Collectively, these findings suggest that dietary interventions targeting glucose availability and metabolic flexibility may exert pleiotropic effects across neurologic, psychiatric, metabolic, and immune systems. While ketogenic diets remain the most extensively studied modality, emerging data indicate that broader VLCD approaches may confer overlapping benefits through shared mechanisms of glucose stabilization, reduced oxidative stress, and immune modulation [67,81,90]. These observations support the use of dietary interventions as adjunctive therapeutic tools for disorders characterized by metabolic instability, while emphasizing the need for individualized application and rigorous clinical evaluation.

## 4. Discussion

### 4.1. Clinical Implications and Future Directions

Emerging evidence suggests that glucose dysregulation can generate inflammatory [93], serologic [94], and psychiatric features [13,14,15,26,45,84,95] that resemble primary autoimmune disease, complicating diagnostic clarity. Because ANA positivity is not highly specific and can arise transiently in metabolic states, refs. [2,40] clinicians risk premature labeling of autoimmune pathology without accounting for underlying glycemic variability. Integrating metabolic evaluation into workups for nonspecific inflammatory symptoms may prevent unnecessary immunosuppression and improve symptom resolution through targeted dietary and lifestyle interventions. Moreover, diagnostic tools such as CGM enable detection of glycemic variability and postprandial glucose excursions before overt diabetes develops [96]. Because these fluctuations can drive inflammatory and immune signaling independently of sustained hyperglycemia, early identification supports a metabolic-first diagnostic approach in select patient populations presenting with autoimmune-mimicking symptoms. Future interdisciplinary efforts integrating endocrinology, immunology, and psychiatry will be necessary to clarify causal pathways, refine screening algorithms, and guide treatment sequencing in patients whose clinical presentations blur traditional metabolic and autoimmune boundaries.

#### 4.1.1. Relevance to Clinical Practice

Incorporating metabolic assessments into standard diagnostic workups is essential for improving diagnostic accuracy and preventing misclassification of autoimmune diseases. Given that transient autoantibody production can arise from impaired apoptotic clearance and metabolic inflammatory signaling [34,42], early screening using CGM, fasting insulin, and glycemic variability indices are feasible and cost-effective additions to clinical practice. Importantly, identifying metabolic contributors early may prevent unnecessary escalation to immunosuppressive or biologic therapies in patients whose symptoms are driven primarily by metabolic rather than immune dysregulation. This approach aligns with precision medicine principles, allowing clinicians to target reversible upstream metabolic dysfunction before labeling patients with chronic autoimmune diagnoses that carry long-term therapeutic and psychosocial consequences.

#### 4.1.2. Continuous Glucose Monitoring in Autoimmunity

Clinically, CGM may provide diagnostic value in patients presenting with nonspecific inflammatory complaints, fatigue, or psychiatric symptoms in whom autoimmune workup is equivocal. CGM could be used in patients with low pretest probability of systemic autoimmune disease to help identify occult glucose dysregulation and guide targeted lifestyle or nutritional interventions prior to labeling disease. Adding CGM to a clinician’s standard autoimmune workup would not only help distinguish between metabolic mimicry and autoimmunity but could also have direct therapeutic implications. For example, patients presenting with fatigue, arthralgia, or rash in the setting of a mildly positive ANA may benefit from an initial period of metabolic stabilization. This aligns with evidence linking metabolic perturbation to elevated ANA prevalence in both type 1 and type 2 diabetes [8]. Additionally, in a study of patients with type 1 diabetes, those with positive autoantibodies had significantly higher hemoglobin A1c levels at around 11.85 compared to 8.52 in auto-antibody-negative patients. There is also a dose-dependent relationship consistent with these elevated findings [97]. Serial reassessment after glucose stabilization may help determine whether ANA titers persist in the absence of metabolic stress, thereby refining diagnostic certainty. This metabolic-first approach is noninvasive, reversible, and aligns with principles of addressing root physiological imbalance.

#### 4.1.3. Future Directions

Despite promising mechanistic links, research gaps remain. Prospective studies are needed to determine whether reducing glycemic variability can normalize nonspecific autoantibody titers or attenuate inflammatory cytokine expression. Controlled dietary intervention trials of the ketogenic diet in a variety of ANA patients should be monitored using CGM to help illuminate causal pathways between glucose dysregulation and immune activation. Because ketosis has demonstrated anti-inflammatory and neuroprotective features [60,95], longitudinal studies should evaluate whether targeted dietary modulation reduces symptom burden in autoimmune-like presentations.

Future research should also explore whether certain CGM patterns (for example: glycemic variability, reactive hypoglycemia, and postprandial spikes) correlate with distinct symptom clusters or biomarker profiles in ANA-positive populations. Additionally, psychiatric manifestations associated with glycemic instability [13,14,15,26] warrant neuropsychiatric outcome tracking in metabolic intervention trials. Finally, integration of CGM-guided dietary modification and metabolic counseling represents a promising multimodal strategy deserving formal clinical study.

## 5. Conclusions

Emerging evidence suggests that glucose dysregulation constitutes a significant but under-recognized driver of inflammatory, serologic, and psychiatric findings that mimic primary autoimmune disease. Glycemic variability, more so than sustained hyperglycemia alone, promotes oxidative stress, mitochondrial dysfunction, and impaired clearance of apoptotic debris. These processes generate transient antibody production and engage inflammatory cascades capable of producing fatigue, arthralgia, rashes, and mood disturbances. As a result, metabolic instability may be misconstrued as early connective tissue disease, prompting unnecessary immunosuppressive therapy or prolonged diagnostic uncertainty.

The convergence of metabolic, immune, and neuroendocrine pathways underscores the necessity of incorporating dynamic metabolic assessments into clinical evaluation. CGM, fasting insulin measurements, and indices of glycemic variability offer feasible avenues for detecting occult dysregulation prior to overt diabetes. Early identification enables targeted dietary and lifestyle interventions that may attenuate autoantibody expression, reduce neuroinflammatory tone, and improve psychiatric symptom burden. Adjunctive strategies such as ketogenic dietary modulation further support autonomic balance and cellular energy efficiency, though larger controlled trials are required to clarify their therapeutic scope.

Despite promising mechanistic links, several knowledge gaps remain. Most evidence derives from small cohorts, cross-sectional analyses, or animal models, limiting causal inference regarding ANA normalization and symptom resolution. Future research should examine whether specific CGM patterns correlate with inflammatory biomarkers, psychiatric outcomes, or autoantibody persistence. Longitudinal studies incorporating dietary modulation and glycemic control will be essential to delineate the extent to which metabolic stabilization can prevent progression to chronic autoimmune pathology.

This review highlights glucose dysregulation as a plausible upstream driver of inflammatory, serologic, and neuropsychiatric features that can mimic autoimmune disease, and recognizing metabolic dysfunction as a potential unifying mechanism broadens the differential diagnosis while inviting a more integrated clinical lens. By accounting for metabolic drivers earlier in evaluation, clinicians may reduce overdiagnosis, minimize unnecessary immunosuppression, and improve patient outcomes through individualized, targeted management. This paradigm highlights metabolism not as a peripheral consideration, but as a central determinant of immune behavior, psychiatric function, and systemic inflammatory tone. Future prospective and interventional studies are essential to determine whether correcting glycemic variability can reverse immune activation and symptomatology, and integrating metabolic assessment into autoimmune evaluation represents a low-risk, high-yield strategy with meaningful implications for patient care.

## Data Availability

See Methods section for detailed analysis of data collection methods. Primary sources included PubMed search results.

## Abbreviations

The following abbreviations are used in this manuscript:

ANA	Anti-Nuclear Antibodies
HPA	Hypothalamus–Pituitary–Adrenal (Axis)
VLCD	Very Low-Carbohydrate Diet
CGM	Continuous Glucose Monitor
CV	Coefficient of Variation
TIR	Time in Range
CRP	C-reactive Protein
ESR	Erythrocyte Sedimentation Rate
NET	Neutrophil Extracellular Traps
NCBI	National Center for Biotechnology Information
PMC	PubMed Central
AGE	Advanced Glycation End Product
HbA1c	Hemoglobin A1c
RAGE	Receptor for Advanced Glycation End Products
8-iso PGF2α	8-isoprostoglandin F2α
Hs-CRP	High-Sensitivity C-Reactive Protein
ROS	Reactive Oxygen Species
TRPA-1	Transient Receptor Potential Analytic-1
CGRP	Calcitonin Gene-Related Peptide
NADPH	Nicotinamide Adenine Dinucleotide Phosphate-Hydrogen
FFA	Free Fatty Acids
NF-kB	Nuclear Factor Kappa-Light Chain Enhancer of Activated B-Cells
CRH	Corticotropin Releasing Hormone
SLE	Systemic Lupus Erythematosus
MCTD	Mixed Connective Tissue Disease
TRPV-1	Transient Receptor Potential Vanilloid-1
